# Undergraduate medical education of Psychiatry in the West

**DOI:** 10.4103/0019-5545.37315

**Published:** 2007

**Authors:** Jolyon T. Dale, Vishal Bhavsar, Dinesh Bhugra

**Affiliations:** Sussex and Brighton Medical School, Brighton, BN1 9PX, UK; * Guys Kings and Thomas Medical School, London SE5, UK; ** Health Service and Population Research Department, Institute of Psychiatry, King's College London, UK

Undergraduate training in Medicine has been through a major structural change in the UK in the past decade or so. The focus on training has been on problem-based learning and is student led. This has raised the profile of some subjects; including Psychiatry, which is now increasingly being used for communication skills training in both primary- and secondary-care settings. In this paper, we illustrate some of the themes as exemplified in the UK training. Some of the special additions are in the field of Special Study Modules which can focus on a number of psychiatric conditions, in addition to the clinical attachments that students are expected to undergo.

Training in Psychiatry at the undergraduate level is crucial for a number of reasons. In the United Kingdom, the prevalence of psychiatric disorders in the community lies at 20%.[1] Of the total population presenting with symptoms in primary care, the prevalence increases to 25%;[1] but amongst general hospital inpatients, the prevalence increases to 40%, with the highest rates amongst Accident and Emergency department admissions. These observations indicate that a high prevalence of mental illness is seen in the United Kingdom. This also means that medical professionals will regularly come into contact with those with mental illness, regardless of their hospital specialties or whether they work in the primary care. These findings are not confined only to the UK. A cross-European study in 2004 showed that 25% of people will suffer from mental illness at some point in their life.[2] In the USA, the national comorbidity survey undertaken between 2000 and 2003 showed that 46.4% of Americans will suffer from mental illness at some stage of their lives.[3]

It therefore makes sense that an understanding of both the diagnosis and management of psychiatric disorders should form an important element of both the teaching and assessment of competence in undergraduate Medicine everywhere. In this paper, we illustrate some of the components from the West, using UK curricula as an example. We also illustrate some of the strengths and weaknesses of the teaching of Psychiatry in undergraduate education in the United Kingdom.

We believe that Psychiatry education in medical schools provides an important avenue for the development of generic “doctoring” skills - such as communication skills, which are at the heart of General Medical Council (GMC), the body responsible for regulating doctors; these expectations are outlined in Good Medical Practice. Also listening, empathy and a holistic approach to patient management can be learnt through Psychiatry and Psychology. Teaching around core common mental disorders such as depression and eating disorders serves as understanding of paradigms of the biopsychosocial model of disease across a number of acute and chronic conditions.

## PSYCHIATRY TEACHING

There are 27 medical schools in the United Kingdom that are recognized by the General Medical Council (GMC).[4] The GMC sets and monitors standards in undergraduate education in the UK and is the main body responsible for assessing and maintaining standards for undergraduate training compared with Post-Graduate Medical Education and Training Board (PMETB).

The core education outcomes, published by the GMC Education Committee in February 2006, recognize that “it is for medical schools to design detailed curricula and schemes of assessment to meet the knowledge, skills, attitudes and behavior that we require of all medical graduates.”[5] Thus there may be some freedom for the medical schools to develop their curricula; it is the GMC which monitors them. There is an ongoing debate as to the need for a generic curricula; the Strategic Options Report recognizes that medical students need to be given a wide range of knowledge, particularly early in their careers as this forms a solid basis for handling clinical situations.[6] The provision of a generic curriculum would also offer clearer career choices for doctors later in their careers. The report recognizes that students should be offered a core curriculum about which they have no choice, noting that mental health, in particular, is often omitted due to a lack of faculty and student commitment. A report from a visit to University of Liverpool Medical School in 2004 outlined concerns over the teaching of mental health. One such concern was that while the level of expectation in student competence was realistic, the curriculum may not be providing an adequate level of preparation for dealing with mental health in the PRHO years.[7]

This is often a common finding across countries as mental illness is seen as lower priority in spite of it being highly prevalent. It may be that stigma plays a role in this, but also it is likely that there are historical reasons for this.

The number of medical students choosing Psychiatry as a career option is rarely above 5% though different medical schools send between 1% and 12% students. Surveys of medical students in the West have suggested that students often view psychiatric patients as especially difficult to deal with.

Despite individual medical schools having some choice over the detailed curricula, the GMC regularly visits and monitors the teaching provided by the constituent medical schools.

One of the key areas of debate in the Strategic Options Consultation was as to whether a standardized national examination should have to be taken by all medical students graduating in the UK. The Consultation found no overall consensus emerging.[6] Many seemed to recognize that more regulation would only be appropriate if there was clear evidence of the need.[7] It is evident that medical education in the UK does enjoy a certain level of diversity between medical schools, and a common exit examination may well provide a clear standard of assessment at a national level.

## SPECIAL STUDY

The length and timing of teaching of Psychiatry varies between medical schools in the UK but always forms a part of undergraduate education. Mental health is rarely covered in detail in the first two years of any medical school education. Rather, many courses, including Oxford and King's College London, contain medical psychology components which assess understanding of psychological models of disease. Other schools have Psychology as an integrated degree in Psychology and similar subjects which may reflect interest in psychosocial matters. However, Student-Selected Components (SSCs) offer medical students the chance to pursue particular areas of interest early on in their study, possibly including areas of mental illness. One such opportunity of an SSC investigating a particular aspect of mental illness is the SSC in “Psychological Aspects of Obesity and Eating Disorders” offered during the “Nutrition, Metabolism and Excretion” module during the first year at Brighton and Sussex Medical School. A particular advantage of the student-selected component to the medical curriculum is the capacity of students to design their own projects in the mental-health arena, offering the opportunity for exploring careers in Psychiatry and developing CVs.

At Brighton and Sussex Medical School, the “Elderly and Mental Health” module in the third year consists of eight weeks of teaching, with half of that dedicated to mental-health teaching. Strong emphasis is put onto multi-professional learning, recognizing the importance of a multi-disciplinary team in clinical practice. Clinical rotations are undertaken in mental health during the third year. Clinical attachments at medical schools in the UK in Psychiatry may be for up to 12 weeks. The assessment at the end of placement includes using Multiple Choice Questions (MCQs); and Observed, Structured, Clinical Examination (OSCE).

Other methods of assessment which may feature throughout medical school education are extended to writing examination questions and essays. In certain medical schools, in the final examination there is one in three chance of getting a psychiatric long case. Final examinations at King's College London Medical School, the largest medical school in the UK, compulsorily contain a psychiatric short case, in which the student is asked to assess a patient with depression, suicidal risk, psychosis, eating disorders, alcohol dependence or posttraumatic stress disorder.

## STRENGTHS

The strengths of the current system are that psychiatry forms an integral part of medical education in every GMC-recognized medical school in the UK. The teaching of Psychiatry guarantees exposure in community, in primary care and in secondary care. As with other areas of undergraduate medical education, clinical experience is important to prepare to enter clinical practice as a qualified professional. Student-Selected Modules (sometimes called Special Study Modules) and Components in Psychiatry and mental health offer detailed learning in undergraduate education which is essentially self-directed, allowing greater preparation for addressing and treating mental health during the foundation years. The GMC has further increased emphasis on the teaching of effective communication skills during undergraduate study. “Tomorrow's Doctors,” which provides recommendations on undergraduate education, states that guidance should be given on how to communicate with those with mental illness, particularly where the patients may have difficulty in sharing their feelings and thoughts with health professionals.[8]

Furthermore, the psychiatric patient population often portrays a starkly different ethnic and sociocultural mix to the general medical and surgical population. We feel that psychiatric rotations within the undergraduate curriculum are a useful way for students to communicate and work with a different patient demographic.

Psychiatric disorders tend to portray a great deal of cultural plasticity; undergraduate experience in Psychiatry can afford students an insight into the way in which illness behaviors of all kinds are culturally constructed.

## WEAKNESSES

There are weaknesses to the undergraduate teaching of Psychiatry. It occupies less time than many other less prevalent medical specializations in undergraduate teaching. Clinical attachments of 6-12 weeks of teaching and attachment out of the 5,500 hours of undergraduate medical study required in the European Union do not adequately reflect the extent of mental illness in the Western world. The focus is invariably put on Psychiatry in secondary care, despite the wide-ranging management of community and primary-care psychiatry. Recent years have seen a sizeable growth in the number of medical students enrolled in undergraduate MB programs in the UK. It is important that the presence of much larger numbers of undergraduate students and consequent increased emphasis on e-learning methods do not compromise the central experience of clinical psychiatric experience - that of direct patient contact, manifested by systematic history taking and mental state examination.

## OBSERVATIONS FROM ELSEWHERE

In Europe teaching standards and objectives differ between countries, although total of at least 5,500 hours of training must be undertaken; most will stipulate that a standard of mental health education must be maintained.

In the United States and Canada, the Liaison Committee on Medical Education (LCME) is recognized as the accrediting authority on awarding an M.D. degree in medical schools. LCME is sponsored by the American Medical Association.[9] A curriculum must be provided that gives a general professional education to prepare for graduate medical teaching. The curriculum should include experiences of care in Psychiatry in using both outpatient and inpatient settings.[10] The importance of care experiences in Psychiatry does not differ between Canada, America and Britain.

## CONCLUSIONS

The teaching of Psychiatry does not often occur until the latter part of undergraduate medical studies. While there is often Psychiatry within the curriculum in the third and fourth years, it does not represent a significant amount; unlike areas such as Cardiology, which may be constantly revisited. Psychiatry seems to be taught in a module, with little links offered to other areas unless it forms an SSC. Offering further time for the teaching of Psychiatry would reflect the impact that mental illness has upon Western society; however, it would mean that other areas of the undergraduate medical curriculum may have to have study time reduced, possible detrimental effects on other areas of care. Changes are likely to occur but not until the positive and negative effects of the recent changes in GMC guidance are fully known. The supervision and monitoring of UK medical schools by the GMC ensures a minimum level of competence in Psychiatry for all junior doctors as they enter F1.
